# Theoretical Study of H/D Isotope Effects on Nuclear Magnetic Shieldings Using an *ab initio* Multi-Component Molecular Orbital Method

**DOI:** 10.3390/molecules18055209

**Published:** 2013-05-07

**Authors:** Taro Udagawa, Takayoshi Ishimoto, Masanori Tachikawa

**Affiliations:** 1 Department of Chemistry and Biomolecular Science, Faculty of Engineering, Gifu University, Yanagido 1-1, Gifu 501-1193, Japan; E-Mail: udagawa@gifu-u.ac.jp; 2 INAMORI Frontier Research Center, Kyushu University, 744 Motooka, Nishi-ku, Fukuoka 819-0395, Japan; E-Mail: ishimoto@ifrc.kyushu-u.ac.jp; 3 Quantum Chemistry Division, Graduate School of Science, Yokohama-city University, Seto 22-2, Kanazawa-ku, Yokohama 236-0027, Japan

**Keywords:** multi-component density functional theory, isotope effect, gauge-including atomic orbital, nuclear magnetic shielding

## Abstract

We have theoretically analyzed the nuclear quantum effect on the nuclear magnetic shieldings for the intramolecular hydrogen-bonded systems of σ-hydroxy acyl aromatic species using the gauge-including atomic orbital technique combined with our multi-component density functional theory. The effect of H/D quantum nature for geometry and nuclear magnetic shielding changes are analyzed. Our study clearly demonstrated that the geometrical changes of hydrogen-bonds induced by H/D isotope effect (called geometrical isotope effect: GIE) is the dominant factor of deuterium isotope effect on ^13^C chemical shift.

## 1. Introduction

Deuterium isotope effect is very important in various fields. For example, kinetic H/D isotope effects are useful to characterize chemical reactions [1], geometrical changes induced by deuterium substitution cause drastic change of the phase transition temperature of hydrogen-bonded dielectric materials [2], and so on. NMR parameters, such as chemical shifts, primary or secondary isotope effects on chemical shifts are used to characterize molecular properties.

**Figure 1:**
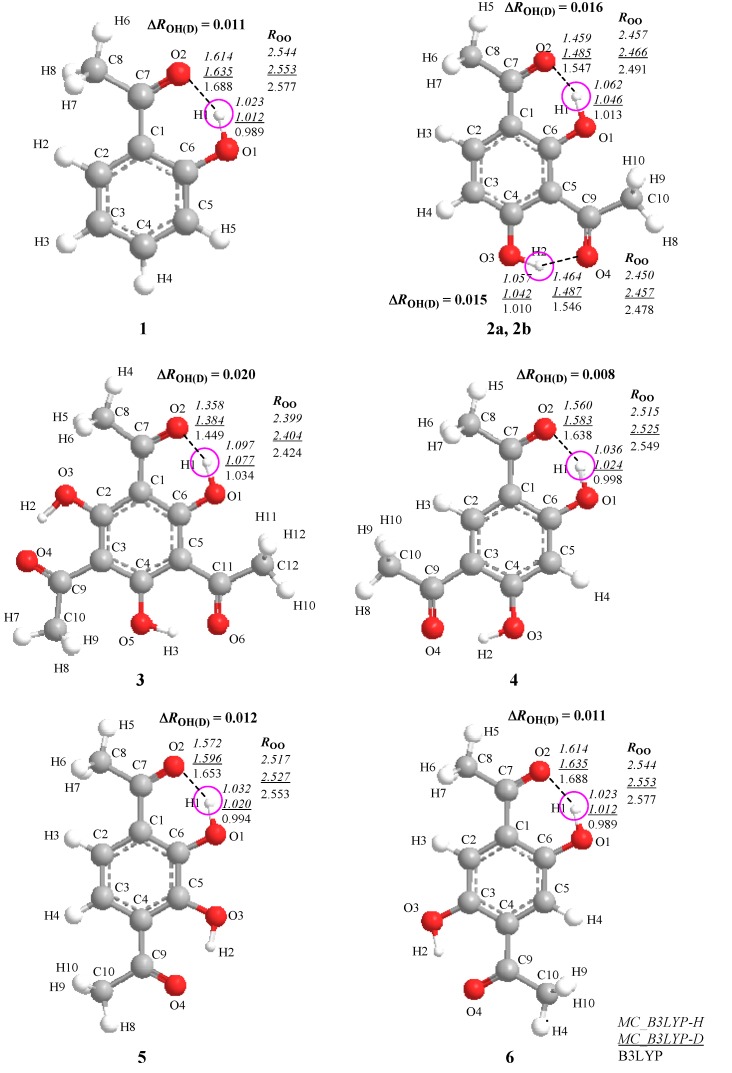
Optimized structures and geometrical parameters (Å) of intramolecular hydrogen-bonded systems.

Recently, Hansen and coworkers [3] investigated deuterium isotope effects on ^13^C chemical shifts for compounds **1**–**6** (Figure 1) both experimentally and theoretically. They chose these six compounds to see the isotope effect on chemical shifts in intramolecular hydrogen-bonded systems depending on the number of neighboring hydrogen-bonded moieties. In their paper, the geometrical differences induced by H/D isotope effect are calculated by fitting the potential to a Morse function. 

We note here that the geometrical H/D isotope effect cannot be directly calculated by only conventional *ab initio* molecular orbital (MO) methods, because only the electronic Schrödinger equation based on the Born-Oppenheimer (BO) approximation [4] is solved in the conventional MO scheme. To represent the geometrical H/D isotope effect within the BO scheme, the BO potential energy surface has to be determined first. Then the geometrical isotope effect (GIE) is obtained from the solution of nuclear Schrödinger equation [5,6]. On the other hands, we have recently developed the multi-component MO (MC_MO) [7,8,9,10] and multi-component density functional theory (MC_DFT) [11,12] methods, which can directly include the nuclear quantum effect and, hence, we can elucidate the difference between quantum nature of proton and deuteron. Using our MC_MO and/or MC_DFT methods, we have already analyzed the GIE of various types of hydrogen-bonds [13,14], kinetic isotope effect in thermal [1,5]-sigmatropic hydrogen shift [15], and so on.

Recently, we have also applied our multi-component method to analyze the nuclear quantum effect and H/D isotope effect on the magnetic properties with the gauge-including atomic orbital (GIAO) [16] and the continuous set of gauge transformation (CSGT) [17] methods [18,19]. Quite recently, Ullah *et al.* have reported deuterium isotope effects on chemical shift of salt-bridged lysines [20]. In their study, isotope effect on nuclear magnetic shieldings is evaluated by performing the Hartree-Fock level of the GIAO calculation at both the MC_MO Hartree-Fock optimized geometry of non-deuterium substituted and deuterium substituted systems. However, there are no reports that analyze the effects of the geometrical differences induced by H/D isotope effects and the nuclear quantum effect on the nuclear magnetic shieldings. 

In this study, thus, we would like to focus on the effect of nuclear quantum effect on the nuclear magnetic shieldings for the compounds **1**–**6** [3] (Figure 1). We will show the main contribution factor of H/D isotope effect on nuclear magnetic shieldings by using GIAO technique combined with MC_DFT method.

## 2. Results and Discussion

### 2.1. Geometrical Changes Induced by H/D Isotope Effect

Figure 1 depicts the optimized geometrical parameters of hydrogen-bond in H and D compounds **1**–**6** according to the multi-component B3LYP (MC_B3LYP) method. Here, only a circled proton/deuteron is treated as quantum wave in the multi-component treatment. Computational details are given in Section 3. In addition, the optimized parameters on each compound with conventional B3LYP are also shown for comparison.

Figure 1 shows that the geometrical changes, induced by the difference between quantum nature of proton and deuteron, can be directly represented by using MC_B3LYP method. The covalent O-H bond length decreases in the order of 
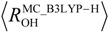
 > 
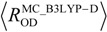
 > 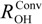, where 
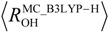
 and 
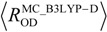
 are the covalent O–H and O–D bond lengths calculated by MC_B3LYP method, respectively, and 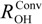 is the equilibrium length with the conventional B3LYP method. This tendency is due to the direct inclusion of the anharmonicity of the potential by MC_DFT method. In addition, the contraction of O–H(D) bond, and the elongation of H(D)^…^O and O^…^O distances by deuterium substitution are clearly found. These changes can be interpreted by using the idea of Ubbelohde effect in hydrogen-bonded crystals [21]. Since these geometrical changes are induced by the difference between quantum nature of proton and deuteron, this geometrical H/D isotope effect is appeared in all the systems studied here.

The changes in the O-H bond length upon deuteration ∆*R*_OH(D)_ are 0.011 Å (**1**), 0.016 Å (**2a**), 0.015 Å (**2b**), 0.020 Å (**3**), 0.008 Å (**4**), 0.012 Å (**5**), and 0.011 Å (**6**), respectively. The magnitudes of ∆*R*_OH(D)_ of **4**–**6** are similar to those in **1** because *R*_OO_ and *R*_H…O_ distances, which represent the strength of hydrogen-bond, in **4**–**6** are very similar to those in **1**. In addition, these values are almost same as those in the previous study of Hansen [3], in which O–H hydrogen displacements are estimated by fitting the potential to a Morse function, 0.0098 Å (**1**), 0.0167 Å (**2a**), 0.0146 Å (**2b**), 0.0211 Å (**3**), 0.0109 Å (**4**), 0.0109 Å (**5**), and 0.0092 Å (**6**).

**Figure 2:**
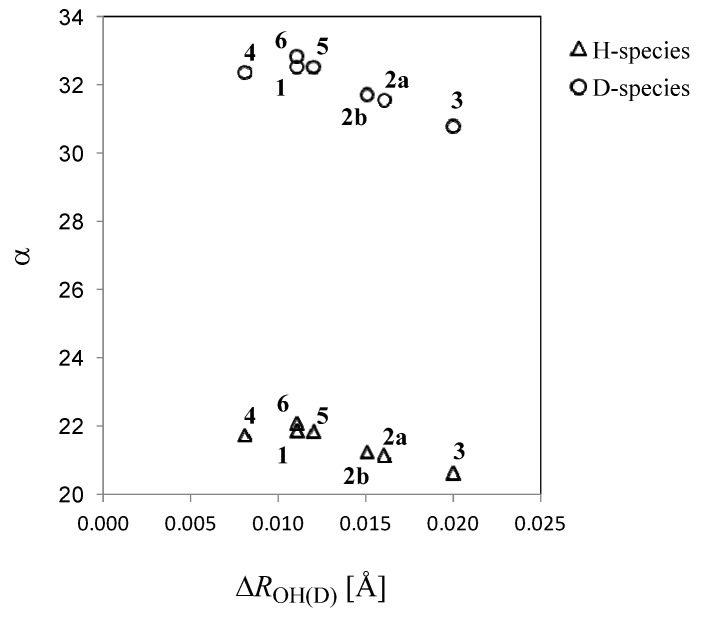
Correlation between the optimized α values and the changes in the *R*_OH_ lengths upon deuteration ∆*R*_OH(D)_.

Next, we focus on the optimized exponent (α) value of GTF as nuclear basis function in each molecule. These values are shown in Table 1. Reflecting a more delocalized proton wavefunction, the optimized α value for a proton is always smaller than that for a deuteron. In addition, the optimized α values for proton and deuteron in compounds **1**–**3** decrease in the order of α(**1**) > α(**2**) > α(**3**). The α values for proton and deuteron in compounds **4**–**6** are very similar to those in **1**, as well as the tendency of ∆*R*_OH(D)_. The optimized α value in GTF correlates with the ∆*R*_OH(D)_ as shown in Figure 2. The smaller α value of **3** than those in other compounds means the small curvature of the potential of **3** due to the strong hydrogen-bonded interaction. Indeed, *R*_OO_ and *R*_H...O_ distances in **3** are significantly shorter than those in other complexes. Thus, the largest ∆*R*_OH(D)_ is found in **3**, although the difference between α(H) and α(D) of **3** is the smallest among **1**–**6**.

**Table 1:** The optimized α value in GTF as the nuclear basis function in each system.

	1	2a	2b	3	4	5	6
H	21.88	21.15	21.27	20.62	21.75	21.85	22.11
D	32.56	31.57	31.71	30.79	32.39	32.54	32.87

### 2.2. Isotope Effects on Nuclear Magnetic Shieldings

The calculated nuclear magnetic shieldings on all atoms in compound **1** are listed in Table 2. The nuclear magnetic shieldings for other compounds are shown in the Supplemenary Information. First, we optimized the H-species (D-species) with the MC_B3LYP method and calculated the values of nuclear magnetic shieldings with MC_B3LYP-GIAO method for the H-species (D-species), which are shown at the second (third) row of MC_B3LYP-H (MC_B3LYP-D) in the Tables. We note here that these values include both the geometrical H/D differences and the nuclear quantum effect. Second, in order to clarify the geometrical effect and nuclear quantum effect on nuclear magnetic shieldings, we have calculated these values by the conventional B3LYP-GIAO method at the MC_B3LYP-optimized geometry of the H-species (D-species), which are shown at the fourth (fifth) row of B3LYP//MC_B3LYP-H (B3LYP//MC_B3LYP-D) in the Tables. We also address here that only the geometrical H/D differences are taken into account in the B3LYP//MC_B3LYP-H and B3LYP//MC_B3LYP-D values. For comparison, the conventional B3LYP-GIAO results at the equilibrium geometry optimized with conventional B3LYP method are shown at the sixth row of B3LYP-Conv. Although Ullah *et al.* [20] have successfully analyzed the isotope effects on nuclear magnetic shieldings by the latter treatment, the influence of the direct quantum-mechanical treatment of proton and deuteron on the isotope effect of nuclear magnetic shielding should be analyzed for a detailed discussion.

**Table 2:** Calculated nuclear magnetic shieldings (ppm) on atoms in **1**.

	MC_B3LYP		B3LYP//MC_B3LYP		B3LYP
	H	D	H	D	Conv
C1	77.02	76.96	76.63	76.49	74.24
C2	66.76	66.72	66.30	66.25	66.19
C3	80.70	80.56	80.21	80.08	79.89
C4	60.95	61.10	60.62	60.73	61.06
C5	77.58	77.75	77.53	77.58	77.73
C6	30.61	31.20	30.43	30.93	32.10
C7	−5.18	−4.97	−5.41	−5.21	−4.64
C8	166.61	166.49	166.22	166.10	165.84
O1	196.05	199.37	194.29	197.87	205.55
O2	−151.26	−158.69	−156.44	−162.30	−174.63
H1	14.86	15.92	16.31	16.96	18.47
H2	24.18	24.18	24.18	24.17	24.17
H3	25.04	25.03	25.03	25.02	25.00
H4	24.34	24.34	24.33	24.33	24.34
H5	24.83	24.83	24.83	24.82	24.82
H6	29.80	29.80	29.81	29.81	29.82
H7	29.06	29.07	29.07	29.07	29.08
H8	29.06	29.07	29.07	29.07	29.08

We focus on the change of nuclear magnetic shieldings from those of the conventional B3LYP calculation. Dramatic changes are found in the nuclear magnetic shieldings on the atoms of quantum-mechanically treated O-H^...^O intramolecular hydrogen-bonds, as was expected. Of course, the nuclear magnetic shielding of the hydrogen atom, which acts as proton donor is drastically changed from 18.47 ppm with conventional B3LYP to 16.96 ppm with deuterium substitution for B3LYP//MC_B3LYP-D, 16.31 ppm for B3LYP//MC_B3LYP-H, 15.92 ppm for MC_B3LYP-D, and 14.86 ppm for MC_B3LYP-H, respectively. The calculated nuclear magnetic shieldings of hydrogen atom in compounds **1**–**6** are shown in Table 3. Reflecting the more delocalized protonic wavefunction due to the strong hydrogen-bonded interaction, the smallest values of nuclear magnetic shielding on hydrogen-bonded proton are found in compound **3**.

**Table 3:** Calculated nuclear magnetic shieldings (ppm) on hydrogen-bonded proton and deuteron in compounds **1**–**6**.

	MC_B3LYP		B3LYP//MC_B3LYP			B3LYP
	H	D	H	D		Conv
1	14.86	15.92	16.31	16.96		18.47
2a	11.90	13.03	13.30	14.03		15.84
2b	12.21	13.30	13.65	14.33		16.03
3	9.68	10.72	11.07	11.72		13.55
4	14.49	15.55	15.95	16.60		18.12
5	14.57	15.68	16.03	16.71		18.31
6	16.11	17.13	17.58	18.18		19.63

The calculated nuclear magnetic shieldings on oxygen atoms, O1 and O2, were also drastically changed from 205.55 and −174.63 ppm with conventional B3LYP to 197.87 and −162.30 ppm by deuterium substitution for B3LYP//MC_B3LYP-D, 194.29 and −156.44 ppm for B3LYP//MC_B3LYP-H, 199.37 and −158.69 ppm for MC_B3LYP-D and 196.05 and −151.26 ppm for MC_B3LYP-H, respectively, because deuterium substitution of hydrogen-bonded proton causes changes in the hydrogen-bonded structure (secondary geometric isotope effect). Large changes of the nuclear magnetic shieldings on carbon atoms are found in C1 and C6, which bond to oxygen atoms. The nuclear magnetic shieldings of C1 and C6 were changed from 74.24 and 32.10 ppm with conventional B3LYP to 76.49 and 30.93 ppm for B3LYP//MC_B3LYP-D, 76.63 and 30.43 ppm for B3LYP//MC_B3LYP-H, 76.96 and 31.20 ppm for MC_B3LYP-D, and 77.02 and 30.61 ppm for MC_B3LYP-H, respectively. Especially, the change on carbon atom bonds to O–H is the deuterium isotope effect on ^13^C chemical shifts, ^2^∆C(OD) and analyzed by Hansen and coworkers [3]. On the other hand, the changes of the nuclear magnetic shieldings on other atoms are extremely small.

Next, we focus on the effect of the direct quantum-mechanical treatment of nuclei on the nuclear magnetic shieldings. This effect can found in the difference between the σ(MC_B3LYP-H) and σ(B3LYP//MC_B3LYP-H). The calculated differences between the σ(MC_B3LYP-H) and σ(B3LYP//MC_B3LYP-H) of hydrogen atom in compounds **1**–**6** are shown in Table 4 (the differences of all atoms in compounds **1**–**6** are shown in the Supplementary Information). This difference of H reflects purely the effect of quantum-mechanical treatment of proton by multi-component procedures, because we used the same geometries for MC_B3LYP-H and B3LYP//MC_B3LYP-H calculations. Of course, the value of D reflects such an effect in deuterium substituted systems. The slight difference of nuclear magnetic shielding between H and D (about 0.4 ppm) indicates the deshielding effect arising from the spatial delocalization from the deuteron to proton, as previously pointed out by Kita *et al*. [18,19].

**Table 4:** Difference between the GIAO-calculated nuclear magnetic shielding (ppm) obtained with the MC_B3LYP and B3LYP//MC_B3LYP methods on hydrogen atom in **1**–**6**.

	H	D
**1**	−1.45	−1.04
**2a**	−1.40	−1.00
**2b**	−1.44	−1.04
**3**	−1.39	−1.00
**4**	−1.46	−1.05
**5**	−1.46	−1.03
**6**	−1.47	−1.05

**Figure 3:**
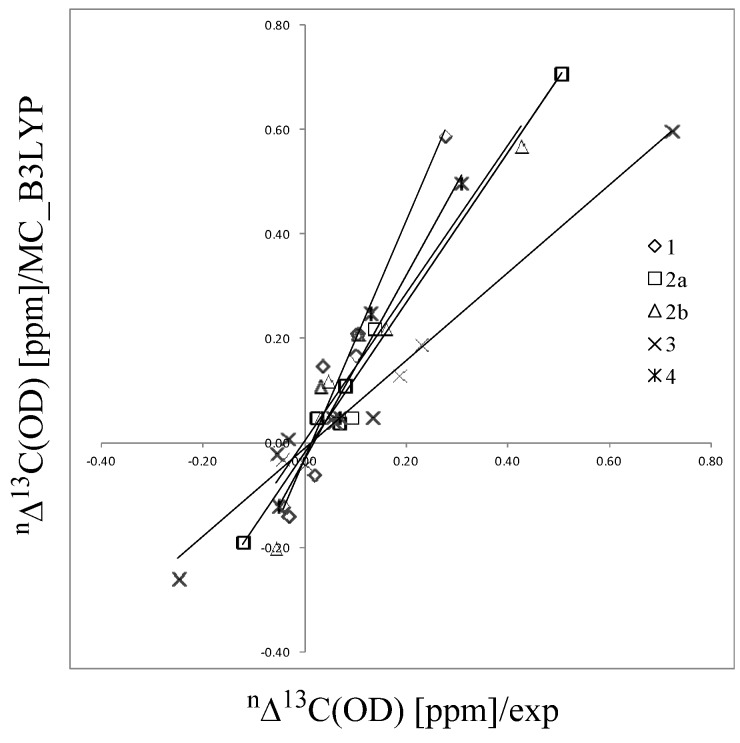
Calculated isotope effects using MC_B3LYP method plotted against the experimental values.

The differences of σ(MC_B3LYP-H) and σ(B3LYP//MC_B3LYP-H) between H- and D-species in compound **1** are listed in Table 5 as σ(MC_B3LYP-H) and σ(B3LYP//MC_B3LYP-H) (these differences for other compounds are shown in the Supplementary Information). It is worth mentioning that ∆σ(MC_B3LYP) includes both the geometrical H/D differences and the nuclear quantum effect, while ∆σ(B3LYP//MC_B3LYP) includes only the former effect. Figure 3 has the experimental ^n^∆C(OD) values, which are defined as the deuterium isotope effects on nuclear magnetic shieldings of carbon atoms ^n^∆C(OD) = σC(D-species) − σC(H-species), where *n* is the intervening number of bonds from the deuterium substitution to the carbon atom, plotted against the ∆σ(MC_B3LYP) values for carbon atoms and the corresponding plot shows good correlation. The calculated nuclear magnetic shieldings are strongly dependent on the calculation method used. Hansen and coworkers have demonstrated that B(PW91)/6-31G(d) GIAO calculations (exchange term only) give reasonable values of nuclear magnetic shieldings [3]. Since only a circled proton/deuteron is treated as a quantum wave, significant differences between σ(MC_B3LYP-H) and σ(B3LYP//MC_B3LYP-H) are only found in quantum treated hydrogen-bonded hydrogens and their neighboring oxygen atoms. The large differences on these atoms clearly demonstrate the influence of the nuclear quantum effect on nuclear magnetic shieldings. Large differences found in neighboring oxygen atoms are caused by deuterium substitution of hydrogen-bonded atom, because deuterium substitution affects the electron distribution around neighboring oxygen atoms. Hansen and coworkers have studied deuterium isotope effects on the ^17^O chemical shifts for compound **3** [22]. Our calculated ^5^∆O(OD) value for compound **3** is −11.25 ppm, which is similar to the experimental ^5^∆^17^O(OD) value, −9.0 ppm.

The conventional B3LYP-GIAO calculation almost represented the H/D isotope effect on the nuclear magnetic shieldings on atoms except for quantum treated hydrogen-bonded hydrogen and neighboring oxygen atoms, if only the optimal structure of H- and D-species were used. The differences between the σ(MC_B3LYP-H) and σ(B3LYP//MC_B3LYP-H) on hydrogen-bonded atoms, which is due to the nuclear quantum effect, are corresponded to 35%–41% of the ∆σ(MC_B3LYP) on hydrogen atom. However, the nuclear quantum effects on ^2^∆C(OD), which is important as a descriptor of hydrogen-bond strength [3], are less than 0.11 ppm for all compounds. These results clearly suggest us that the GIE induced by deuterium substitution is the dominant factor of the deuterium isotope effect on nuclear magnetic shieldings because deuterium isotope effects on nuclear magnetic shieldings can be reproduced qualitatively by the conventional B3LYP-GIAO calculations at the MC_B3LYP-optimized geometry of H- and D-species. 

**Table 5:** Difference between the calculated nuclear magnetic shielding ∆σ (ppm) induced by H/D isotope effect on atoms in **1**.

	MC_B3LYP ^a^	B3LYP//MC_B3LYP ^a^
C1	−0.06	−0.14
C2	−0.04	−0.05
C3	−0.14	−0.13
C4	0.15	0.11
C5	0.17	0.05
C6	0.59	0.50
C7	0.21	0.20
C8	−0.21	−0.12
O1	3.32	3.58
O2	−7.43	−5.86
H1	1.06	0.65
H2	0.00	−0.01
H3	−0.01	−0.01
H4	0.00	0.00
H5	0.00	−0.01
H6	0.00	0.00
H7	0.01	0.00
H8	0.01	0.00

^a^ The calculated H/D isotope effect on nuclear shieldings using the MC_B3LYP-GIAO method and using the conventional B3LYP-GIAO//MC_B3LYP method are defined as ∆σ(MC_B3LYP) = σ(MC_B3LYP-D) − σ(MC_B3LYP-H) and ∆σ(B3LYP//MC_B3LYP) = σ(B3LYP//MC_B3LYP-D) − σ(B3LYP//MC_B3LYP-H), respectively.

To summarize, the origin of deuterium substitution on nuclear magnetic shielding is the difference of quantum nature between proton and deuteron. Basically, the conventional B3LYP-GIAO calculations using the optimized geometry of H- and D-species by MC_B3LYP can qualitatively reproduce the experimental deuterium isotope effect on nuclear magnetic shielding. The direct inclusion of nuclear quantum effect is required for quantitative discussions. 

## 3. Theory and Computational Details

To directly describe the H/D geometrical isotope effects, we use the multi-component hybrid density functional theory (MC_DFT). In our MC_DFT approach, the Kohn-Sham operators for electrons and quantum nuclei are derived by adding the hybrid-type exchange-correlation potentials to Fock operators of Hartree-Fock level of multi-component MO method as:

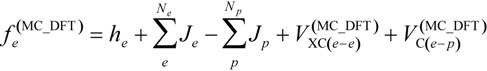
(1)

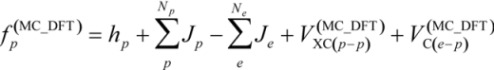
(2)
where 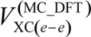 is given as:

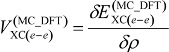
(3)


To improve accuracy of calculations, many-body effects such as electron-electron correlation should be considered by appropriate procedures. In this study, only electron-electron correlation is considered with the aid of the B3LYP exchange-correlation functional [23]. Strictly speaking, in the framework of the multi-component treatment, new types of many-body effects such as electron-nucleus and nucleus-nucleus correlations [11,24] should be taken into account to improve the accuracy. However, in this stage, we have ignored these correlations due to its small contribution to the total energy. More detailed information of our MC_DFT method is published elsewhere [11,12]. 

In the framework of multi-component calculations, some light nuclei such as protons and deuterons can be treated as quantum particles. Here, only one of the intramolecular hydrogen-bonded protons (circled in Figure 1) is treated quantum-mechanically, and the other nuclei are treated as point charges. To evaluate the nuclear magnetic shieldings, the GIAO technique is combined with the MC_DFT [18,19] method. First, to analyze the effect of geometrical changes induced by the H/D isotope effect on nuclear magnetic shieldings, non-deuterium substituted (H-species) and deuterium substituted species (D-species) are optimized by the multi-component B3LYP (MC_B3LYP) method. The 6-31G** basis set and single *s*-type GTF are adopted as electronic and nuclear basis sets, respectively, and the exponent values of nuclear single *s*-type GTFs are optimized. Second, the GIAO technique combined with conventional B3LYP (B3LYP-GIAO) and MC_B3LYP (MC_B3LYP-GIAO) calculations are performed on optimized geometry of H- and D-species, to clarify main contribution factors of the H/D isotope effect on nuclear magnetic shieldings. 

We have calculated differently substituted 2-hydroxyacetophenones, such as 2'-hydroxyacetophenone (**1**), 1,3-diacetyl-2,4-dihydroxybenzene (**2**), 1,5-diacetyl-2,4-dihydroxybenzene (**4**), 1,4-diacetyl-2,3-dihydroxybenzene (**5**), and 1,4-diacetyl-2,5-dihydroxybenzene (**6**), and 1,3,5-triacetyl-2,4,6-trihydroxybenzene (**3**), as shown in Figure 1 [3], as the models of intramolecular hydrogen-bonded systems. All calculations were performed with modified version of GAUSSIAN03 program package [25].

## 4. Conclusions

Nuclear magnetic shieldings were calculated with the conventional B3LYP and the multi-component B3LYP (MC_B3LYP) methods based on the optimized geometries obtained under MC_B3LYP. We obtained different hydrogen-bond structures in H- and D-compounds due to the direct treatment of the H/D nuclear quantum effects. We clearly demonstrated that the GIE is the dominant factor of H/D isotope effect on nuclear magnetic shieldings. For more detailed discussion for the H/D difference of nuclear magnetic shieldings, direct treatment of H/D quantum effect is important under the GIAO calculation.

## Supplementary Materials

Supplementary materials can be accessed at: http://www.mdpi.com/1420-3049/18/5/5209/s1.
