# Supplementary material for: Theoretical Study of H/D Isotope Effects on Nuclear Magnetic Shieldings Using an ab initio Multi-Component Molecular Orbital Method
Source: Molecules. 2013 May 7;18(5):5209–20. doi: 10.3390/molecules18055209 (PMC6269809; doi:10.3390/molecules18055209)
Supplement: Supplementary file 1 [file molecules-18-05209-s001.pdf]

# Supplementary Materials

**Table S1.** Calculated nuclear magnetic shieldings (ppm) on atoms in **2a**.

| | MC_B3LYP | | B3LYP//MC_B3LYP | | B3LYP |
|---|---|---|---|---|---|
| | **H** | **D** | **H** | **D** | **Conv** |
| C1 | 83.89 | 83.93 | 83.53 | 83.48 | 83.38 |
| C2 | 60.48 | 60.45 | 60.01 | 59.97 | 59.90 |
| C3 | 87.33 | 87.29 | 86.86 | 86.82 | 86.79 |
| C4 | 24.23 | 24.45 | 23.88 | 24.07 | 24.55 |
| C5 | 84.45 | 84.56 | 83.98 | 84.04 | 84.15 |
| C6 | 24.83 | 25.54 | 24.66 | 25.29 | 26.74 |
| C7 | −2.39 | −2.34 | −2.74 | −2.67 | −2.31 |
| C8 | 167.32 | 167.13 | 166.93 | 166.73 | 166.33 |
| C9 | −6.00 | −6.00 | −6.26 | −6.28 | −6.26 |
| C10 | 158.82 | 158.80 | 158.49 | 158.46 | 158.37 |
| O1 | 170.04 | 175.24 | 168.62 | 174.02 | 185.54 |
| O2 | −99.37 | −109.21 | −103.97 | −112.57 | −131.02 |
| O3 | 174.77 | 175.49 | 174.39 | 175.06 | 176.87 |
| O4 | −143.19 | −144.31 | −143.41 | −144.46 | −147.36 |
| H1 | 11.90 | 13.03 | 13.30 | 14.03 | 15.84 |
| H2 | 15.85 | 15.87 | 15.82 | 15.87 | 16.03 |
| H3 | 24.23 | 24.23 | 24.24 | 24.23 | 24.21 |
| H4 | 25.51 | 25.50 | 25.50 | 25.49 | 24.47 |
| H5 | 29.81 | 29.81 | 29.82 | 29.82 | 29.83 |
| H6 | 29.19 | 29.19 | 29.19 | 29.19 | 29.19 |
| H7 | 29.19 | 29.19 | 29.19 | 29.19 | 29.19 |
| H8 | 29.65 | 29.65 | 29.65 | 29.64 | 29.64 |
| H9 | 28.82 | 28.82 | 28.81 | 28.82 | 28.82 |
| H10 | 28.82 | 28.82 | 28.81 | 28.82 | 28.82 |

**Table S2.** Calculated nuclear magnetic shieldings (ppm) on atoms in **2b**.

| | MC_B3LYP | | B3LYP//MC_B3LYP | | B3LYP |
|---|---|---|---|---|---|
| | **H** | **D** | **H** | **D** | **Conv** |
| C1 | 84.49 | 84.32 | 83.83 | 83.67 | 83.38 |
| C2 | 59.98 | 60.09 | 59.55 | 59.64 | 59.90 |
| C3 | 86.54 | 86.75 | 86.44 | 86.54 | 86.79 |
| C4 | 23.03 | 23.60 | 22.81 | 23.32 | 24.55 |
| C5 | 84.92 | 84.89 | 84.53 | 84.41 | 84.15 |
| C6 | 26.82 | 26.94 | 26.41 | 26.50 | 26.74 |
| C7 | −1.81 | −1.88 | −2.41 | −2.21 | −2.31 |
| C8 | 166.74 | 166.73 | 166.37 | 166.35 | 166.33 |
| C9 | −6.86 | −6.64 | −7.12 | −6.90 | −6.26 |
| C10 | 159.38 | 159.18 | 159.00 | 158.80 | 158.37 |
| O1 | 184.47 | 184.90 | 184.05 | 184.44 | 185.54 |
| O2 | −126.97 | −128.06 | −127.26 | −128.25 | −131.02 |
| O3 | 160.93 | 166.21 | 160.34 | 165.57 | 176.87 |
| O4 | −118.39 | −127.33 | −123.03 | −130.72 | −147.36 |

**Table S2.** *Cont.*

| | MC_B3LYP | | B3LYP//MC_B3LYP | | B3LYP |
|---|---|---|---|---|---|
| | H | D | H | D | Conv |
| H1 | 15.64 | 15.69 | 15.65 | 15.69 | 15.84 |
| H2 | 12.21 | 13.30 | 13.65 | 14.33 | 16.03 |
| H3 | 24.22 | 24.21 | 24.22 | 24.21 | 24.21 |
| H4 | 25.48 | 25.48 | 25.48 | 25.48 | 24.47 |
| H5 | 29.83 | 29.83 | 29.83 | 29.83 | 29.83 |
| H6 | 29.20 | 29.20 | 29.20 | 29.20 | 29.19 |
| H7 | 29.20 | 29.20 | 29.20 | 29.20 | 29.19 |
| H8 | 29.62 | 29.63 | 29.63 | 29.63 | 29.64 |
| H9 | 28.80 | 28.81 | 28.82 | 28.82 | 28.82 |
| H10 | 28.80 | 28.81 | 28.82 | 28.82 | 28.82 |

**Table S3.** Calculated nuclear magnetic shieldings (ppm) on atoms in **3**.

| | MC_B3LYP | | B3LYP//MC_B3LYP | | B3LYP |
|---|---|---|---|---|---|
| | H | D | H | D | Conv |
| C1 | 89.94 | 90.04 | 89.78 | 89.78 | 89.60 |
| C2 | 21.35 | 21.48 | 20.94 | 21.06 | 21.35 |
| C3 | 90.61 | 90.54 | 89.80 | 89.73 | 89.77 |
| C4 | 21.09 | 21.28 | 20.77 | 20.93 | 21.34 |
| C5 | 89.70 | 89.87 | 89.18 | 89.31 | 89.63 |
| C6 | 19.68 | 20.28 | 19.40 | 19.95 | 21.38 |
| C7 | −5.68 | −5.63 | −6.07 | −6.01 | −5.58 |
| C8 | 160.09 | 159.83 | 159.75 | 159.47 | 158.82 |
| C9 | −5.22 | −5.26 | −5.52 | −5.56 | −5.63 |
| C10 | 159.30 | 159.27 | 158.98 | 158.95 | 158.83 |
| C11 | −5.35 | −5.34 | −5.61 | −5.60 | −5.60 |
| C12 | 159.33 | 159.31 | 158.94 | 159.91 | 158.88 |
| O1 | 139.56 | 146.82 | 138.70 | 146.07 | 162.30 |
| O2 | −73.53 | −84.78 | −77.04 | −87.55 | −111.25 |
| O3 | 161.05 | 161.51 | 160.63 | 161.06 | 162.27 |
| O4 | −107.31 | −108.25 | −107.62 | −108.52 | −111.18 |
| O5 | 160.38 | 160.97 | 160.01 | 160.58 | 162.26 |
| O6 | −106.76 | −107.90 | −107.05 | −108.15 | −111.22 |
| H1 | 9.68 | 10.72 | 11.07 | 11.72 | 13.55 |
| H2 | 13.37 | 13.42 | 13.36 | 13.40 | 13.55 |
| H3 | 13.36 | 13.40 | 13.36 | 13.40 | 13.54 |
| H4 | 29.66 | 29.66 | 29.67 | 29.67 | 29.67 |
| H5 | 28.85 | 28.85 | 28.86 | 28.86 | 28.86 |
| H6 | 28.85 | 28.85 | 29.68 | 28.86 | 28.86 |
| H7 | 29.68 | 29.68 | 29.68 | 29.68 | 29.67 |
| H8 | 28.87 | 28.87 | 28.86 | 28.86 | 28.86 |
| H9 | 28.87 | 28.87 | 28.86 | 28.86 | 28.86 |
| H10 | 29.69 | 29.69 | 29.68 | 29.67 | 29.68 |
| H11 | 28.86 | 28.86 | 28.85 | 28.85 | 28.86 |
| H12 | 28.86 | 28.86 | 28.85 | 28.85 | 28.86 |

**Table S4.** Calculated nuclear magnetic shieldings (ppm) on atoms in **4**.

| | **MC_B3LYP** | | **B3LYP//MC_B3LYP** | | **B3LYP** |
|---|---|---|---|---|---|
| | **H** | **D** | **H** | **D** | **Conv** |
| C1 | 82.61 | 82.60 | 83.23 | 82.14 | 81.98 |
| C2 | 61.19 | 61.22 | 60.74 | 60.75 | 60.74 |
| C3 | 82.63 | 82.62 | 82.00 | 81.98 | 81.98 |
| C4 | 27.45 | 27.51 | 27.02 | 27.07 | 27.25 |
| C5 | 91.43 | 91.48 | 91.27 | 91.22 | 91.13 |
| C6 | 26.01 | 26.51 | 25.79 | 26.22 | 27.25 |
| C7 | −2.91 | −2.66 | −3.13 | −2.89 | −2.25 |
| C8 | 167.34 | 167.22 | 166.96 | 166.84 | 166.60 |
| C9 | −1.91 | −1.95 | −2.21 | −2.24 | −2.25 |
| C10 | 166.98 | 166.97 | 166.60 | 166.60 | 166.60 |
| O1 | 178.79 | 182.49 | 177.39 | 181.27 | 189.71 |
| O2 | −142.69 | −149.84 | −147.59 | −153.28 | −165.38 |
| O3 | −164.94 | −164.93 | −164.75 | −164.75 | −165.38 |
| O4 | 189.55 | 189.68 | 188.97 | 189.11 | 189.71 |
| H1 | 14.49 | 15.55 | 15.95 | 16.60 | 18.12 |
| H2 | 18.07 | 18.07 | 18.07 | 18.06 | 18.12 |
| H3 | 23.88 | 23.89 | 23.89 | 23.89 | 23.88 |
| H4 | 25.53 | 25.53 | 25.53 | 25.52 | 25.50 |
| H5 | 29.78 | 29.79 | 29.80 | 29.80 | 29.80 |
| H6 | 29.15 | 29.16 | 29.17 | 29.17 | 29.17 |
| H7 | 29.15 | 29.16 | 29.17 | 29.17 | 29.17 |
| H8 | 29.81 | 29.81 | 29.80 | 29.81 | 29.80 |
| H9 | 29.18 | 29.18 | 29.17 | 29.17 | 29.17 |
| H10 | 29.18 | 29.18 | 29.17 | 29.17 | 29.17 |

**Table S5.** Calculated nuclear magnetic shieldings (ppm) on atoms in **5**.

| | **MC_B3LYP** | | **B3LYP//MC_B3LYP** | | **B3LYP** |
|---|---|---|---|---|---|
| | **H** | **D** | **H** | **D** | **Conv** |
| C1 | 74.97 | 74.85 | 74.47 | 74.29 | 73.99 |
| C2 | 80.22 | 80.15 | 79.73 | 79.66 | 79.56 |
| C3 | 80.62 | 80.42 | 80.15 | 79.97 | 79.56 |
| C4 | 74.59 | 74.59 | 73.88 | 73.87 | 73.99 |
| C5 | 38.52 | 38.69 | 38.27 | 38.38 | 38.67 |
| C6 | 36.69 | 37.43 | 36.66 | 37.28 | 38.67 |
| C7 | −5.98 | −5.77 | −6.21 | −6.00 | −5.44 |
| C8 | 166.10 | 165.96 | 165.63 | 165.49 | 165.30 |
| C9 | −5.25 | −5.23 | −5.46 | −5.45 | −5.44 |
| C10 | 165.64 | 165.65 | 165.28 | 165.29 | 165.30 |
| O1 | 199.93 | 203.79 | 198.59 | 202.59 | 211.07 |
| O2 | −158.82 | −167.00 | −164.21 | −170.79 | −184.48 |
| O3 | 210.30 | 210.62 | 210.20 | 210.41 | 211.07 |
| O4 | −183.45 | −183.65 | −183.36 | −183.53 | −184.07 |

**Table S5.** *Cont.*

| | MC_B3LYP | | B3LYP//MC_B3LYP | | B3LYP |
|---|---|---|---|---|---|
| | **H** | **D** | **H** | **D** | **Conv** |
| H1 | 14.57 | 15.68 | 16.03 | 16.71 | 18.31 |
| H2 | 18.19 | 18.21 | 18.19 | 18.20 | 18.31 |
| H3 | 24.84 | 24.84 | 24.83 | 24.83 | 24.82 |
| H4 | 24.87 | 24.86 | 24.86 | 24.85 | 24.82 |
| H5 | 29.73 | 29.74 | 29.76 | 29.74 | 29.75 |
| H6 | 29.09 | 29.10 | 29.09 | 29.09 | 29.11 |
| H7 | 29.09 | 29.10 | 29.09 | 29.09 | 29.11 |
| H8 | 29.75 | 29.75 | 29.75 | 29.75 | 29.75 |
| H9 | 29.11 | 29.11 | 29.10 | 29.10 | 29.11 |
| H10 | 29.11 | 29.11 | 29.10 | 29.10 | 29.11 |

**Table S6.** Calculated nuclear magnetic shieldings (ppm) on atoms in **6**.

| | MC_B3LYP | | B3LYP//MC_B3LYP | | B3LYP |
|---|---|---|---|---|---|
| | **H** | **D** | **H** | **D** | **Conv** |
| C1 | 71.89 | 71.80 | 71.48 | 71.32 | 71.20 |
| C2 | 77.52 | 77.40 | 76.92 | 76.80 | 76.49 |
| C3 | 40.31 | 42.53 | 42.11 | 41.96 | 41.80 |
| C4 | 71.08 | 71.25 | 70.60 | 70.73 | 71.20 |
| C5 | 76.62 | 76.79 | 76.43 | 76.48 | 76.49 |
| C6 | 40.31 | 40.90 | 40.17 | 40.65 | 41.80 |
| C7 | −6.83 | −6.57 | −7.03 | −6.79 | −6.08 |
| C8 | 165.98 | 165.88 | 165.73 | 165.63 | 165.35 |
| C9 | −6.12 | −6.07 | −6.35 | −6.31 | −6.08 |
| C10 | 165.63 | 165.64 | 165.41 | 165.42 | 165.35 |
| O1 | 215.66 | 218.22 | 213.60 | 216.52 | 223.06 |
| O2 | −180.50 | −187.09 | −185.77 | −190.71 | −201.89 |
| O3 | 224.32 | 223.98 | 223.53 | 223.25 | 223.06 |
| O4 | −202.49 | −201.99 | −201.87 | −201.45 | −201.89 |
| H1 | 16.11 | 17.13 | 17.58 | 18.18 | 19.63 |
| H2 | 19.56 | 19.54 | 19.56 | 19.54 | 19.63 |
| H3 | 24.64 | 24.64 | 24.63 | 24.62 | 24.64 |
| H4 | 24.62 | 24.63 | 24.62 | 24.62 | 24.64 |
| H5 | 29.73 | 29.74 | 29.74 | 29.74 | 29.75 |
| H6 | 29.01 | 29.02 | 29.02 | 29.02 | 29.03 |
| H7 | 29.01 | 29.02 | 29.02 | 29.02 | 29.03 |
| H8 | 29.75 | 29.75 | 29.75 | 29.75 | 29.75 |
| H9 | 29.02 | 29.03 | 29.02 | 29.02 | 29.03 |
| H10 | 29.02 | 29.03 | 29.02 | 29.02 | 29.03 |

**Table S7.** Differences between the GIAO-calculated nuclear magnetic shieldings (ppm) obtained with the MC_B3LYP and B3LYP//MC_B3LYP methods on atoms in each molecule.

| | **1** | | **2a** | | **2b** | | **3** | | **4** | | **5** | | **6** | |
|---|---|---|---|---|---|---|---|---|---|---|---|---|---|---|
| | **H** | **D** | **H** | **D** | **H** | **D** | **H** | **D** | **H** | **D** | **H** | **D** | **H** | **D** |
| C1 | 0.39 | 0.47 | 0.36 | 0.45 | 0.66 | 0.65 | 0.16 | 0.26 | −0.62 | 0.46 | 0.50 | 0.56 | 0.41 | 0.48 |
| C2 | 0.46 | 0.47 | 0.47 | 0.48 | 0.43 | 0.45 | 0.41 | 0.42 | 0.45 | 0.47 | 0.49 | 0.49 | 0.60 | 0.60 |
| C3 | 0.49 | 0.48 | 0.47 | 0.47 | 0.10 | 0.21 | 0.81 | 0.81 | 0.63 | 0.64 | 0.47 | 0.45 | −1.80 | 0.57 |
| C4 | 0.33 | 0.37 | 0.35 | 0.38 | 0.22 | 0.28 | 0.32 | 0.35 | 0.43 | 0.44 | 0.71 | 0.72 | 0.48 | 0.52 |
| C5 | 0.05 | 0.17 | 0.47 | 0.52 | 0.39 | 0.48 | 0.52 | 0.56 | 0.16 | 0.26 | 0.25 | 0.31 | 0.19 | 0.31 |
| C6 | 0.18 | 0.27 | 0.17 | 0.25 | 0.41 | 0.44 | 0.28 | 0.33 | 0.22 | 0.29 | 0.03 | 0.15 | 0.14 | 0.25 |
| C7 | 0.23 | 0.24 | 0.35 | 0.33 | 0.33 | 0.33 | 0.39 | 0.38 | 0.22 | 0.23 | 0.23 | 0.23 | 0.20 | 0.22 |
| C8 | 0.39 | 0.39 | 0.39 | 0.40 | 0.37 | 0.38 | 0.34 | 0.36 | 0.38 | 0.38 | 0.47 | 0.47 | 0.25 | 0.25 |
| C9 | | | 0.26 | 0.28 | 0.26 | 0.26 | 0.30 | 0.30 | 0.30 | 0.29 | 0.21 | 0.22 | 0.23 | 0.24 |
| C10 | | | 0.33 | 0.34 | 0.38 | 0.38 | 0.32 | 0.32 | 0.38 | 0.37 | 0.36 | 0.36 | 0.22 | 0.22 |
| C11 | | | | | | | 0.26 | 0.26 | | | | | | |
| C12 | | | | | | | 0.39 | −0.60 | | | | | | |
| O1 | 1.76 | 1.50 | 1.42 | 1.22 | 0.42 | 0.46 | 0.86 | 0.75 | 1.40 | 1.22 | 1.34 | 1.20 | 2.06 | 1.70 |
| O2 | 5.18 | 3.61 | 4.60 | 3.36 | 0.29 | 0.19 | 3.51 | 2.77 | 4.90 | 3.44 | 5.39 | 3.79 | 5.27 | 3.62 |
| O3 | | | 0.38 | 0.43 | 0.59 | 0.64 | 0.42 | 0.45 | −0.19 | −0.18 | 0.10 | 0.21 | 0.79 | 0.73 |
| O4 | | | 0.22 | 0.15 | 4.64 | 3.39 | 0.31 | 0.27 | 0.58 | 0.57 | −0.09 | −0.12 | −0.62 | −0.54 |
| O5 | | | | | | | 0.37 | 0.39 | | | | | | |
| O6 | | | | | | | 0.29 | 0.25 | | | | | | |
| H1 | −1.45 | −1.04 | −1.4 | −1.00 | −0.01 | 0.00 | −1.39 | −1.00 | −1.46 | −1.05 | −1.46 | −1.03 | −1.47 | −1.05 |
| H2 | 0.00 | 0.01 | 0.00 | 0.00 | −1.44 | −1.03 | 0.01 | 0.02 | 0.00 | 0.01 | 0.00 | 0.01 | 0.00 | 0.00 |
| H3 | 0.01 | 0.01 | −0.01 | 0.00 | 0.00 | 0.00 | 0.00 | 0.00 | −0.01 | 0.00 | 0.01 | 0.01 | 0.01 | 0.02 |
| H4 | 0.01 | 0.01 | 0.01 | 0.01 | 0.00 | 0.00 | −0.01 | −0.01 | 0.00 | 0.01 | 0.01 | 0.01 | 0.00 | 0.01 |
| H5 | 0.00 | 0.01 | 0.00 | 0.00 | 0.00 | 0.00 | −0.01 | −0.01 | −0.02 | −0.01 | −0.01 | 0.00 | −0.01 | 0.00 |
| H6 | −0.01 | −0.01 | 0.00 | 0.00 | 0.00 | 0.00 | −0.01 | −0.01 | −0.02 | −0.01 | 0.00 | 0.01 | −0.01 | 0.00 |
| H7 | −0.01 | 0.00 | 0.00 | 0.00 | 0.00 | 0.00 | 0.00 | 0.00 | −0.02 | −0.01 | 0.00 | 0.01 | −0.01 | 0.00 |
| H8 | −0.01 | 0.00 | 0.00 | 0.01 | −0.01 | 0.00 | 0.01 | 0.01 | 0.01 | 0.00 | 0.00 | 0.00 | 0.00 | 0.00 |
| H9 | | | 0.01 | 0.00 | −0.02 | −0.01 | 0.01 | 0.01 | 0.01 | 0.01 | 0.01 | 0.01 | 0.00 | 0.01 |
| H10 | | | 0.01 | 0.00 | −0.02 | −0.01 | 0.01 | 0.02 | 0.01 | 0.01 | 0.01 | 0.01 | 0.00 | 0.01 |
| H11 | | | | | | | 0.01 | 0.01 | | | | | | |
| H12 | | | | | | | 0.01 | 0.01 | | | | | | |

**Table S8.** Differences between the calculated nuclear magnetic shieldings Δσ (ppm) induced by H/D isotope effect on atoms in compounds **2–6**.

| | **2a** | | **2b** | | **3** | | **4** | | **5** | | **6** | |
|---|---|---|---|---|---|---|---|---|---|---|---|---|
| | MC_B3LYP [a] | B3LYP//MC_B3LYP [a] | MC_B3LYP [a] | B3LYP//MC_B3LYP [a] | MC_B3LYP [a] | B3LYP//MC_B3LYP [a] | MC_B3LYP [a] | B3LYP//MC_B3LYP [a] | MC_B3LYP [a] | B3LYP//MC_B3LYP [a] | MC_B3LYP [a] | B3LYP//MC_B3LYP [a] |
| C1 | 0.04 | −0.05 | −0.17 | −0.16 | 0.10 | 0.00 | −0.01 | −1.09 | −0.12 | −0.18 | −0.09 | −0.16 |
| C2 | −0.03 | −0.04 | 0.11 | 0.09 | 0.13 | 0.12 | 0.03 | 0.01 | −0.07 | −0.07 | −0.12 | −0.12 |
| C3 | −0.04 | −0.04 | 0.21 | 0.10 | −0.07 | −0.07 | −0.01 | −0.02 | −0.20 | −0.18 | 2.22 | −0.15 |
| C4 | 0.22 | 0.19 | 0.57 | 0.51 | 0.19 | 0.16 | 0.06 | 0.05 | 0.00 | −0.01 | 0.17 | 0.13 |
| C5 | 0.11 | 0.06 | −0.03 | −0.12 | 0.17 | 0.13 | 0.05 | −0.05 | 0.17 | 0.11 | 0.17 | 0.05 |
| C6 | 0.71 | 0.63 | 0.12 | 0.09 | 0.60 | 0.55 | 0.50 | 0.43 | 0.74 | 0.62 | 0.59 | 0.48 |
| C7 | 0.05 | 0.07 | −0.07 | −0.07 | 0.05 | 0.06 | 0.25 | 0.24 | 0.21 | 0.21 | 0.26 | 0.24 |
| C8 | −0.19 | −0.20 | −0.01 | −0.02 | −0.26 | −0.28 | −0.12 | −0.12 | −0.14 | −0.14 | −0.10 | −0.10 |
| C9 | 0.00 | −0.02 | 0.22 | 0.22 | −0.04 | −0.04 | −0.04 | −0.03 | 0.02 | 0.01 | 0.05 | 0.04 |
| C10 | −0.02 | −0.03 | −0.20 | −0.20 | −0.03 | −0.03 | −0.01 | 0.00 | 0.01 | 0.01 | 0.01 | 0.01 |
| C11 | | | | | 0.01 | 0.01 | | | | | | |
| C12 | | | | | −0.02 | 0.97 | | | | | | |
| O1 | 5.20 | 5.40 | 0.43 | 0.39 | 7.26 | 7.37 | 3.70 | 3.88 | 3.86 | 4.00 | 2.56 | 2.92 |
| O2 | −9.84 | −8.60 | −1.09 | −0.99 | −11.25 | −10.51 | −7.15 | −5.69 | −8.18 | −6.58 | −6.59 | −4.94 |
| O3 | 0.72 | 0.67 | 5.28 | 5.23 | 0.46 | 0.43 | 0.01 | 0.00 | 0.32 | 0.21 | −0.34 | −0.28 |
| O4 | −1.12 | −1.05 | −8.94 | −7.69 | −0.94 | −0.90 | 0.13 | 0.14 | −0.20 | −0.17 | 0.50 | 0.42 |
| O5 | | | | | 0.59 | 0.57 | | | | | | |
| O6 | | | | | −1.14 | −1.10 | | | | | | |
| H1 | 1.13 | 0.73 | 0.05 | 0.04 | 1.04 | 0.65 | 1.06 | 0.65 | 1.11 | 0.68 | 1.02 | 0.60 |
| H2 | 0.05 | 0.05 | 1.09 | 0.68 | 0.05 | 0.04 | 0.00 | −0.01 | 0.02 | 0.01 | −0.02 | −0.02 |
| H3 | 0.00 | −0.01 | −0.01 | −0.01 | 0.04 | 0.04 | 0.01 | 0.00 | 0.00 | 0.00 | 0.00 | −0.01 |
| H4 | −0.01 | −0.01 | 0.00 | 0.00 | 0.00 | 0.00 | 0.00 | −0.01 | −0.01 | −0.01 | 0.01 | 0.00 |
| H5 | 0.00 | 0.00 | 0.00 | 0.00 | 0.00 | 0.00 | 0.01 | 0.00 | 0.01 | 0.00 | 0.01 | 0.00 |
| H6 | 0.00 | 0.00 | 0.00 | 0.00 | 0.00 | 0.00 | 0.01 | 0.00 | 0.01 | 0.00 | 0.01 | 0.00 |
| H7 | 0.00 | 0.00 | 0.00 | 0.00 | 0.00 | 0.00 | 0.01 | 0.00 | 0.01 | 0.00 | 0.01 | 0.00 |
| H8 | 0.00 | −0.01 | 0.01 | 0.00 | 0.00 | 0.00 | 0.00 | 0.01 | 0.00 | 0.01 | 0.00 | 0.00 |
| H9 | 0.00 | 0.01 | 0.01 | 0.00 | 0.00 | 0.00 | 0.00 | 0.00 | 0.00 | 0.00 | 0.01 | 0.00 |
| H10 | 0.00 | 0.01 | 0.01 | 0.00 | 0.00 | −0.01 | 0.00 | 0.00 | 0.00 | 0.00 | 0.01 | 0.00 |
| H11 | | | | | 0.00 | 0.00 | | | | | | |
| H12 | | | | | 0.00 | 0.00 | | | | | | |

[a] The calculated H/D isotope effect on nuclear shieldings using the conventional B3LYP-GIAO//MC_B3LYP method and using the MC_B3LYP-GIAO method are defined as Δσ(B3LYP//MC_B3LYP) = σ(B3LYP//MC_B3LYP-D) − σ(B3LYP//MC_B3LYP-H) and Δσ(MC_B3LYP) = σ(MC_B3LYP-D) − σ(MC_B3LYP-H), respectively.
